# The role of Raptor in lymphocytes differentiation and function

**DOI:** 10.3389/fimmu.2023.1146628

**Published:** 2023-05-22

**Authors:** Jianing Tang, Lu Yang, Fei Guan, Heather Miller, Niels Olsen Saraiva Camara, Louisa K. James, Kamel Benlagha, Masato Kubo, Steffen Heegaard, Pamela Lee, Jiahui Lei, Hu Zeng, Chengwei He, Zhimin Zhai, Chaohong Liu

**Affiliations:** ^1^ Department of Pathogen Biology, School of Basic Medicine, Tongji Medical College and State Key Laboratory for Diagnosis and Treatment of Severe Zoonostic Infectious Disease, Huazhong University of Science and Technology, Wuhan, Hubei, China; ^2^ Cytek Biosciences, R&D Clinical Reagents, Fremont, CA, United States; ^3^ Department of Immunology, Institute of Biomedical Sciences, University of São Paulo (USP), São Paulo, SP, Brazil; ^4^ Centre for Immunobiology, Bizard Institute, Queen Mary University of London, London, United Kingdom; ^5^ Université de Paris, Institut de Recherche Saint-Louis, EMiLy, Paris, France; ^6^ Laboratory for Cytokine Regulation, Center for Integrative Medical Science (IMS), Rikagaku Kenkyusho, Institute of Physical and Chemical Research (RIKEN) Yokohama Institute, Yokohama, Japan; ^7^ Department of Ophthalmology, Rigshospitalet Glostrup, Copenhagen, Denmark; ^8^ Department of Clinical Medicine, University of Copenhagen, Copenhagen, Denmark; ^9^ Department of Paediatrics and Adolescent Medicine, Li Ka Shing Faculty of Medicine, The University of Hong Kong, Hong Kong, Hong Kong SAR, China; ^10^ Department of Immunology, Mayo Clinic, Rochester, MN, United States; ^11^ Division of Rheumatology, Department of Medicine, Mayo Clinic, Rochester, MN, United States; ^12^ State Key Laboratory of Quality Research in Chinese Medicine, Institute of Chinese Medical Sciences, University of Macau, Taipa, Macao SAR, China; ^13^ Department of Hematology, The Second Hospital of Anhui Medical University, Hefei, China

**Keywords:** Raptor, mTORC1, T cell, B cell, immune

## Abstract

Raptor, a key component of mTORC1, is required for recruiting substrates to mTORC1 and contributing to its subcellular localization. Raptor has a highly conserved N-terminus domain and seven WD40 repeats, which interact with mTOR and other mTORC1-related proteins. mTORC1 participates in various cellular events and mediates differentiation and metabolism. Directly or indirectly, many factors mediate the differentiation and function of lymphocytes that is essential for immunity. In this review, we summarize the role of Raptor in lymphocytes differentiation and function, whereby Raptor mediates the secretion of cytokines to induce early lymphocyte metabolism, development, proliferation and migration. Additionally, Raptor regulates the function of lymphocytes by regulating their steady-state maintenance and activation.

## Background

Mammalian target of rapamycin (mTOR) is a serine/threonine protein kinase that belongs to the PI3K-related kinase (PIKK) family. mTOR mediates phosphorylation of eukaryotic initiation factor 4E-binding protein 1 (4E-BP1) and p70 S6 kinase (p70S6k), which is essential in regulating daily metabolism, including mediating protein synthesis, cell differentiation, aging, and autophagy (1–4). The phosphatidylinositol-3-kinase (PI3K)-Akt-mTOR signaling pathway is a core pathway in many forms of human cancers (5), in which it is usually activated at abnormal levels. mTOR's role in cancer demonstrates its importance as a therapeutic target for cancer treatment (6–9). However, mTOR plays a vital role in normal physiological conditions as well, where it participates and mediates basic cell metabolism and differentiation (10).

The discovery of mTOR dates back to the 1960s. Its discovery came after that of rapamycin, which was identified as the mammalian target of Rapamycin (mTOR). Rapamycin, discovered in 1962, inhibits the autoimmune response and suppresses tumor growth (11). Rapamycin interacts with mTORC1 (12) and is an inhibitor of Raptor-mTORC1. Rapamycin can bind FKBP12 and form the complex, rapamycin–FKBP12, which is a specific inhibitor of Raptor- mTORC1 (13). The mTOR-FKBP12 complex, discovered in 1992, mediates mTOR anti-proliferative functions (14), and was identified in mammalian cells by genetic screening for Rapamycin-resistance in 1994 (15).

**Figure 1:**
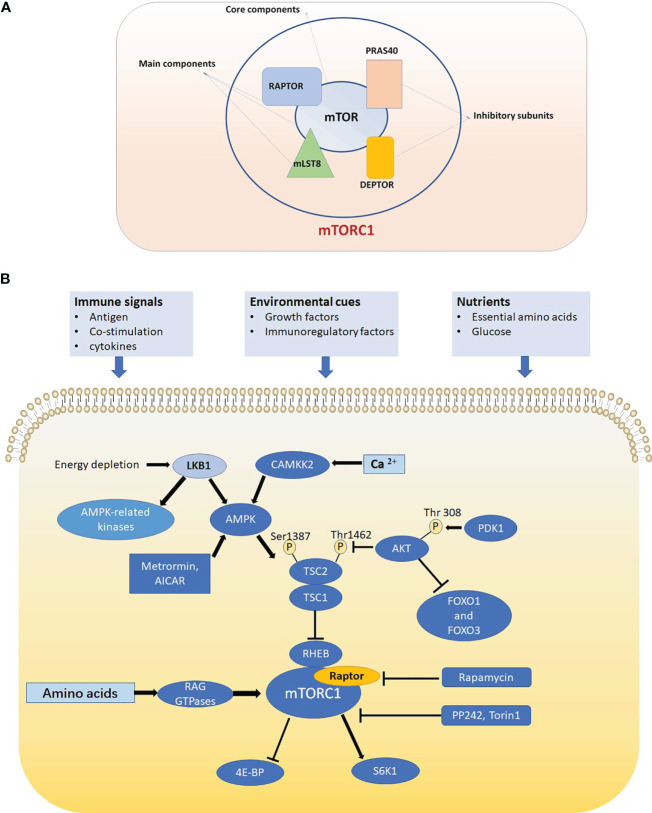
**(A)** mTORC1 is composed of mTOR, Raptor, mLST8, PRAS40 and DEPTOR. mTOR is the core component of mTORC1. Raptor and mLST8 are the two other main components and PRAS40 DEPTOR are two inhibitory subunits of mTORC1. **(B)** In T cell, mTORC1 is sensitive to three kinds of signals including immune signals, nutrients and environmental cues and mTORC1 can promote translation initiation and protein synthesis by directly phosphorylating ribosomal protein S6 kinases (S6Ks) and eIF4E-binding proteins (4E-BPs). Raptor-mTORC1 signaling pathway play crucial roles in cell signaling, metabolism and autophagy.

mTOR has two protein complexes and acts as a core component in the mTOR complex 1 (mTORC1) and mTOR complex 2 (mTORC2), which have different functions and are characterized by two different proteins: regulatory-associated protein of mTOR (Raptor) and rapamycin-insensitive companion of mTOR (Rictor) (1, 16). Since mTORC1 but not mTORC2 can be specifically inhibited by rapamycin, most studies have focused on mTORC1 and its role in metabolism. Previous studies have demonstrated mTORC1 as a central regulator of anabolism and catabolism, which features responding to nutrition condition (4). The function of mTORC1 is more focused on cell growth and survival, and mTORC2 is more inclined to cell proliferation and cytoskeletal remodeling. However, deciphering the mechanism of the function of mTORC2 is more challenging for lack of specific pharmacological inhibitors (17). mTORC1 is composed of three main components: mTOR (core component), Raptor (a regulatory protein), mLST8 (mammalian lethal with sec13 protein 8) (10, 18, 19) and two inhibitory subunits: PRAS40 (20), and DEPTOR (21) (**Figure 1A**).

Raptor, a crucial part of mTORC1, recruits substrates to mTORC1 by binding to specific canonical substrates, which contributes to the subcellular localization of mTORC1 (22, 23). Raptor has a conserved domain in the N-terminus and seven WD40 repeats. This distinctive domain is expected to contribute to the interactions with mTOR and mTORC1-associated proteins (1, 19). The official name of Raptor gene is RPTOR, KOG1 as well as Mip1, and the gene ID is 57521. The human RPTOR gene locates in Chromosome 17 and starts from 80544838 to 80966368. The gene structure of mTORC1 and other related molecules has been clearly summarized by Tatebe, H. and Shiozaki, K (24). Growth factors, amino acids, lipids and cholesterol regulate the function of mTORC1 and lysosomes are the primary target (25, 26).

Growth factors such as epidermal growth factor (EGF) and insulin-like growth factor (IGF) bind to their receptors to mediate mTORC1-related signaling pathways like the PI3K-PDK1-Akt signaling pathway. As a core part of mTORC1, Raptor is also a target, which when phosphorylated, activates mTORC1 (27). For example, the activation of the mitogen-activated protein kinase (MAPK) pathway, ERK/RSK/ICK, leads to phosphorylation of Raptor, which activates mTORC1 (28–30). Raptor mediates mTORC1 functions such as cell metabolism, which consequently, affects cell growth and development. There are many studies demonstrating that Raptor/mTORC1 regulates lymphocyte function and differentiation through control of basic metabolism and finally influences the immunity (31, 32). The role of Raptor is different in various kinds of lymphocytes, and this review we systematically summarize the role of Raptor in them.

## Raptor’s role in B cells

The developmental process of B cells is tightly regulated, which has many checkpoints during gene arrangement, positive selection and negative selection (31, 33). Looking at the expression of immunoglobulin chains and some cell surface proteins, the development and maturation process of B cells can be divided into the following stages: pre-pro B cell stage, pro B cell stage, early pre B cell stage, late pre B cell stage, immature and mature B cell stage, and the developing positions (BM or fetal liver) of B cell. If errors occur in the differentiation process, clonal deletion will happen (16, 34, 35). In order to avoid this interference when studying the function of Raptor-mTORC1, researchers knocked out the Raptor gene at specific stages. They found that Raptor-mTORC1 not only affects early B cell differentiation stages, but also B cells after the pre-B cell stage. Raptor was found to have many critical roles, including promoting B cells to produce Ig H chain (IgH), contributing to the maintenance of pre-B cell homeostasis and survival, and regulating the intracellular respiration and glycolysis of B cells to promote antibody production (16). In B-cells, Raptor regulates B cell differentiation and function, although it is more critical for regulating differentiation (36). Raptor impacts the differentiation process via cellular bioenergetics in B cells (16, 34). Interleukin-7 (IL–7) is an essential cytokine for B cell maturation (37, 38) and deletion of Raptor causes a decline in the production of IL–7, which causes a blockage in B cell development.

## Raptor mediates the transition from pro-B cells to pre-B cells in an IL-7R signaling and Myc expression dependent way

**Figure 2:**
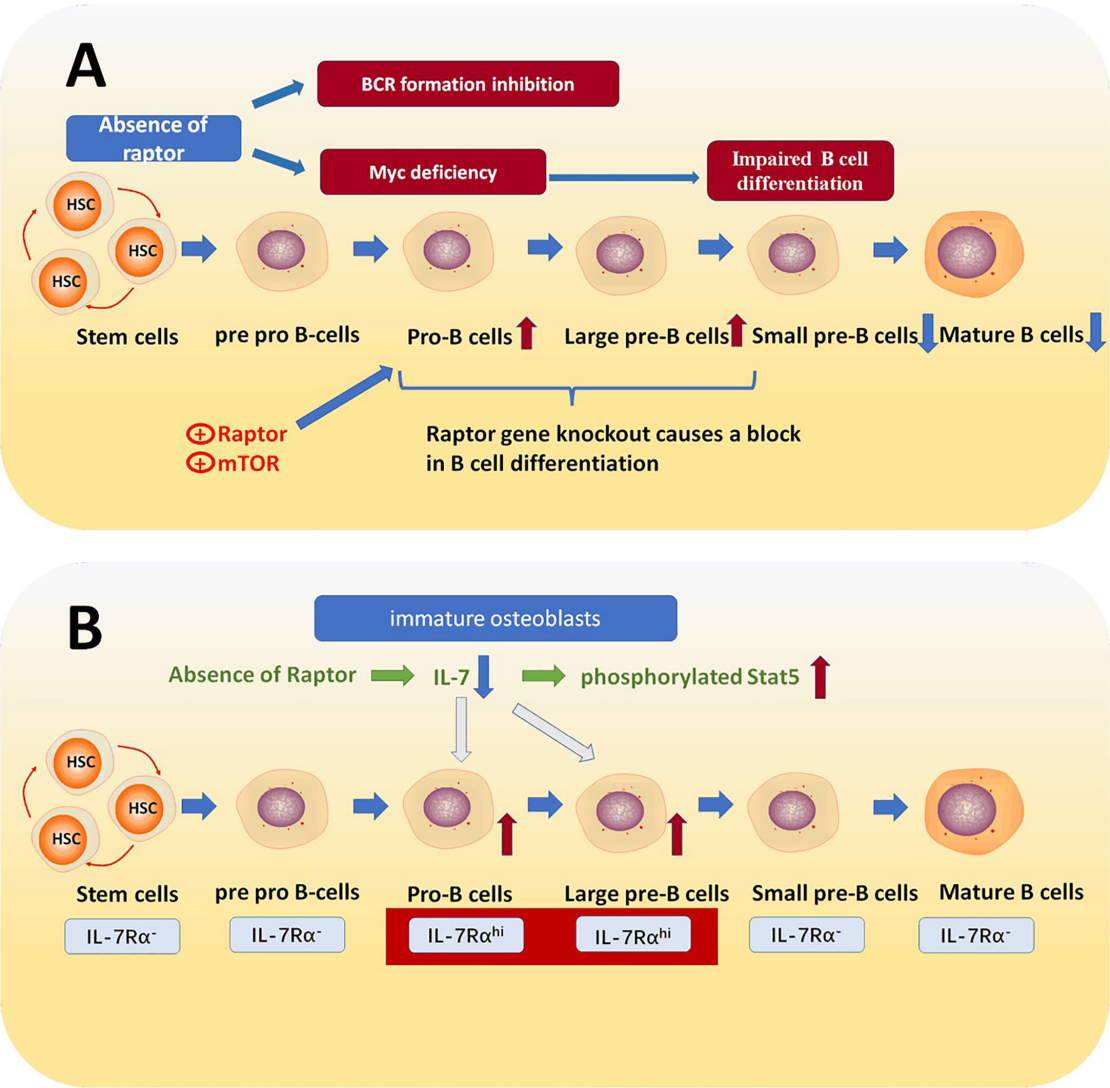
**(A)** Raptor mediates the transition from pro B cells to pre-B cells. The absence of raptor causes the inactivation of mTORC1, which is a downstream effector of IL-7R signaling and essential for Myc expression and consequently blocks the transition from pro-B cells to pre-B cells. **(B)** IL-7 receptors are increased in pro-B cell and large pre B cell stages, which is essential for B cell fate. IL-7 is a crucial cytokine in B cell differentiation process, and the absence of Raptor down-regulates the expression of IL-7 in immature osteoblasts, which leads to abnormal IL-7/Stat5 signaling and thereby prevents pro B cells transforming into pre B cells.

By using Raptor^fl/fl^ mice, and crossing Raptor^fl/fl^ mice with transgenic Mb1-Cre mice to generate B cell lineage Raptor deletion mice, researchers examined the expression levels of mTORC1 pathway associated signaling proteins including Raptor, B220, CD43, HAS, BP1, IgH2, and Ig L chain (IgL) in KO and WT mouse bone marrow. They also aimed to determine the necessary expression conditions in the WT mouse group. The final result was that the expression level of Raptor was higher in large and small pre-B cells, while lower in small dormant immature and mature B cells. Not only the expression of proteins in mTORC1 related signaling pathway such as mTOR and Raptor is at higher levels, but also downstream proteins in the mTORC1 signaling pathway, p-S6R and p-4EBP1, are expressed at higher levels. These results suggest that in pro B and pre B cell stages, the mTORC1 signaling pathway and the expression of Raptor is more active. The reduced Raptor level in the late B cell stages corresponded with IgM and IgD expression (16). Looking further into the increased expression of Raptor in pro B to pre B stages by examining the expression of related proteins in WT and RaptorKO mice revealed that in B cell progenitors of RaptorKO mice, Raptor, mTOR, p-S6K1, p-S6R, and p-4EBP1 all had a significant decrease compared to the WT group. This strongly suggests that the lack of Raptor greatly disrupts the mTORC1 signaling pathway. The researchers also used flow cytometry and other techniques to detect the number of B cells in the RaptorKO group, and found that immature and mature B cells in the bone marrow of the RaptorKO group were largely absent relative to the WT group. Also, it was found that in peripheral tissues, there were decreased quantities of B220+ splenic B cells, B2 cells, and CD5+IgM+ B1a cells (16). Overall, Raptor plays an important role in B cell differentiation and the production of B cell surface markers; and the deficiency of Raptor causes a block in pro B to pre B stages. To explain this further, based on the fact that the proto-oncogene Myc is essential for B cell development (39–41), mTORC1 plays an irreplaceable role in IL-7 induced Myc expression in pro-B cells (42) (**Figure 2A**). Other studies also demonstrated that deficiency of Raptor inhibits early B cells’ ability to properly respond to early BCR stimulations or express BCR (16), thus, the deficiency of Raptor inhibits the B cell development process.

## Raptor in immature osteoblasts blocks IL- 7 expression and then mediates B cell differentiation

B cells develop and mature in bone marrow (BM), so the bioactive components in the BM microenvironment impacts B cell differentiation (43). Furthermore, it has been already shown that mTORC1 regulates cell metabolism and proliferation as well as differentiation. The blockage of the mTORC1 signaling pathway in bone lineage cells causes reduced bone production in mice (44), and it has also been shown that Raptor deficiency in some osteolineage cells affects B cell differentiation (36). Moreover, early osteoblasts can produce C-X-C motif chemokine 12 (CXCL12) and IL-7, which are necessary in regulating B cell differentiation (45–47).

IL-7 is a crucial cytokine for various signaling pathways. For example, STAT5a/b, PI3 Kinase, STAT5 and SRC kinases can all be activated by IL-7, which is essential for immunity (48). Relevant studies have shown that IL-7 is necessary for the development and maturation of lymphocytes and that IL-7 mediates B cell development via activation of stromal derived factor1 (SDF1), and fms-related tyrosine kinase 3 ligand (FLT3LG) (49, 50). Osterix is a zinc finger-containing transcription factor proved to be essential for osteoblast differentiation (51). Studies have found that Raptor deficiency in Osterix-expressing cells leads to a decreased number of total B cells and suppresses pro-B cell transform into pre-B cells. Furthermore, it was also detected that the apoptosis rate of pre B cells is abnormally higher in Raptor deficiency mice than WT (36). In all, the deficiency of Raptor causes a block in the mTORC1 signaling pathway and inhibits IL-7 expression. Despite other pathways related to IL-7, what has been demonstrated is that Raptor deficiency in mice causes an increased level of phosphorylated Stat5 in pro B cells, which prevents development into pre B cells and then into immature B cells (36, 52, 53) (**Figure 2B**).

Interestingly, after providing RaptorKO mice with recombinant murine IL-7, the quantity of B cells in all developmental stages is close to normal (36, 54). Additionally, although CXCL12 is essential for B cell maturation and is expressed by osterix-expressing cells too, the quantity of CXCL12 is unaffected under the condition of Raptor gene specific knockout (36).

## The role of raptor in T cells

Originating from the bone marrow and mature in the thymus, T cell is an essential part of the immune system to every Individual organism (55). In the thymus, T cells multiply and transit into helper, regulatory, cytotoxic, and memory T cells (56, 57). The transition process can be mediated by multiple factors and one of the factors is mTOR signaling pathway, which is sensitive to three kinds of signals including immune signals, nutrients and environmental cues (4, 58, 59) (**Figure 1B**). T cells are more dependent on Raptor than B cells. Both CD 4+, CD 8+ subsets and γδ, αβ subsets are regulated by Raptor (60). According to relevant studies, mTOR related signaling pathways regulate T cells’ differentiation and function by mediate their metabolic programing, which has intimate connections with membrane molecules expression and macromolecule synthesis (4, 61–63). CD4+ (helper) T cells play an irreplaceable role in immunity. T helper (Th) cells produce crucial cytokines that activate cytotoxic T cells in response to specific immunological challenges (64). Additionally, the cytokines produced by the Th lineage cells are crucial for immunity (65). As with other lymphocytes, mTORC1 can also regulates T helper cell metabolism, which including mediates the expression of glycolytic enzymes in these cells, thereby influencing their proliferation and function (66). It has been discovered that inhibiting mTOR with rapamycin influences naïve T cells to transform into Th1, Th2, and Th17 cells etc (67–69).

## Raptor inhibits memory CD8 T cell differentiation via downstream effectors like S6K1 and eIF4E

**Figure 3:**
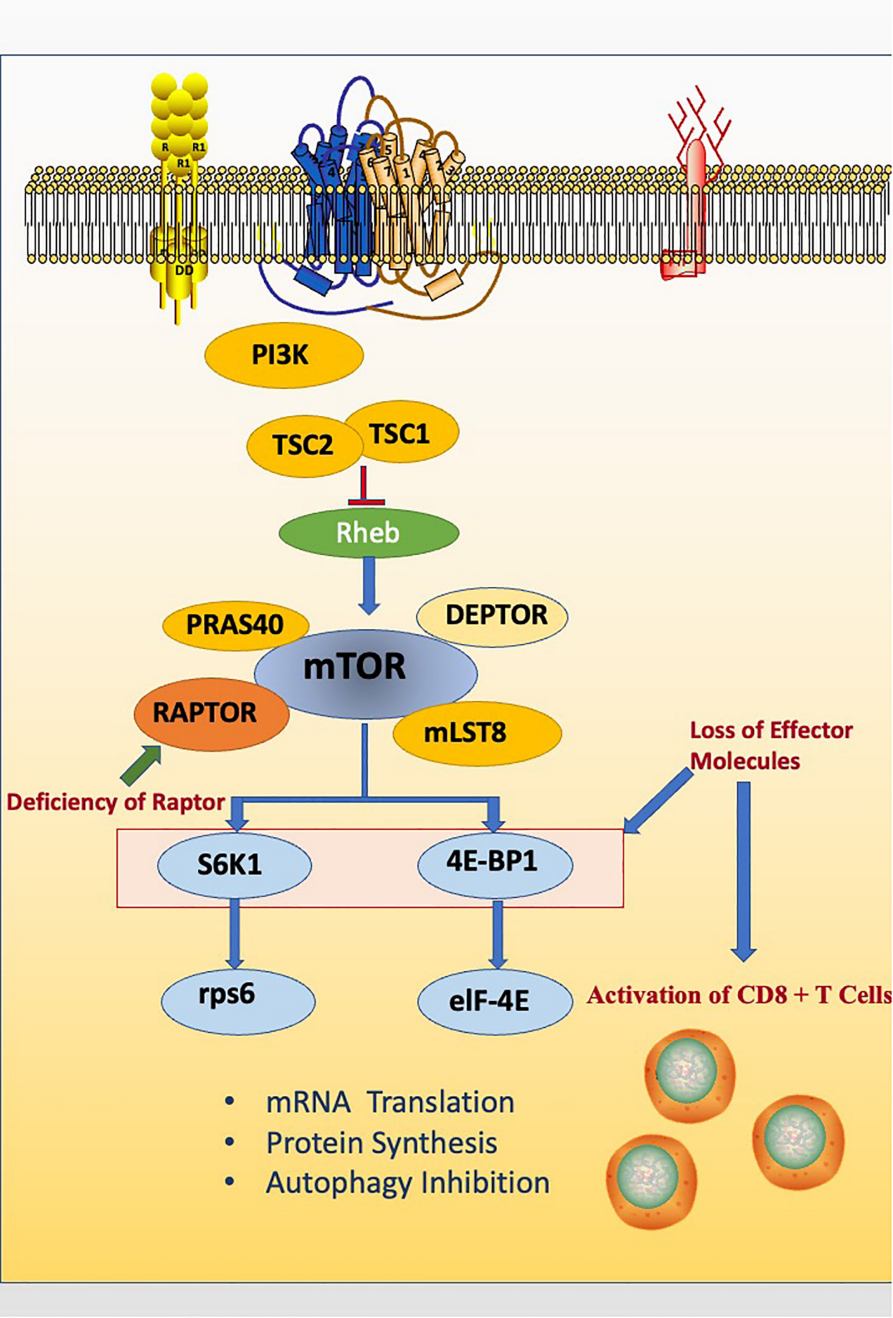
Raptor inhibits memory CD8 T cell differentiation via downstream effectors like S6K1 and eIF4E. The deficiency of Raptor leads to the abnormal activation of CD8 + T cells.

CD8+ T cells, also known as cytotoxic T lymphocytes (CTLs), are the core component of cellular immunity. Thus, the activation of CD8 + T cells is the aim of improving many vaccines (70). Rapamycin can suppress interleukin-2-stimulated T cell proliferation. Thus, it is widely used to treat autoimmune diseases, particularly after organ transplantation (3, 12, 71). In a surprising study, after treating acute lymphocytic choriomeningitis virus infected mice with rapamycin, it was found that the amount of virus-specific CD8+ T cells increased. This phenomenon was undoubtedly controversial to previous perceptions. In this study, four biomarkers were used to testify the quantity and quality of memory CD 8 + T cells in rapamycin treated mice, including CD127, CD62L, KLRG1 and Bcl2, which are all relevant to long-lived protective immunity (72, 73). In order to explain this abnormal phenomenon, researchers specifically knocked down Raptor gene using a retrovirus based RNA interference (RNAi) system and demonstrated that rapamycin essentially inhibits memory CD8 T cell differentiation. While particularly in memory CD8 T cells, there is an unusual inhibition of the differentiation process caused by Raptor-mTORC1 signaling pathway intrinsically (60). Researchers finally found that the mechanism of Raptor-mTORC1 signaling pathway inhibition is via S6K1 and eIF4E, which are mTORC1 downstream effectors that down-regulate memory CD 8 + T cell differentiation (**Figure 3**) (60).

Additionally, other researchers reported that rapamycin can also suppress the proliferation of memory CD8 + T cells in intestinal and vaginal mucosa. Over all, these findings found both a new role of Raptor and rapamycin in lymphocyte functions (74).

## WAVE2 maintains T cell homeostasis and function via binding to Raptor and mTOR

By regulating TCR-stimulated actin cytoskeletal dynamics, WASp family verprolin homologous protein 2 (WAVE2) can mediates T cell functions (75). According to some research, WAVE2 is expressed more in hematopoietic cells and the deficiency of WAVE2 causes abnormal activation of T cells. It has also been reported that WAVE2-related signaling pathways play crucial roles in the invasion process and metastasis keeping of cancer cells and may be a novel target for cancer therapy in future (76–78). Scientists have found that WAVE 2 KO mice have severe autoimmune disease and inflammatory response compared to the WT controls (79, 80). The WAVE KO mice suffered from splenomegaly, lymphadenopathy, multi-organ lymphocytic infiltration, and died early. It was also found that AKT-mTOR activation and metabolic reprogramming are dysregulated in WAVE 2 KO mice, which leads to overactivation of T cell and autoimmune disease. Furthermore, WAVE2 also acts as a signal coupling regulator between the antigens and cytokines of T cells. The compromised antigen-specific T cell immune response and greater apoptosis rate of T cells also occurs in WAVE 2 KO mice.

**Figure 4:**
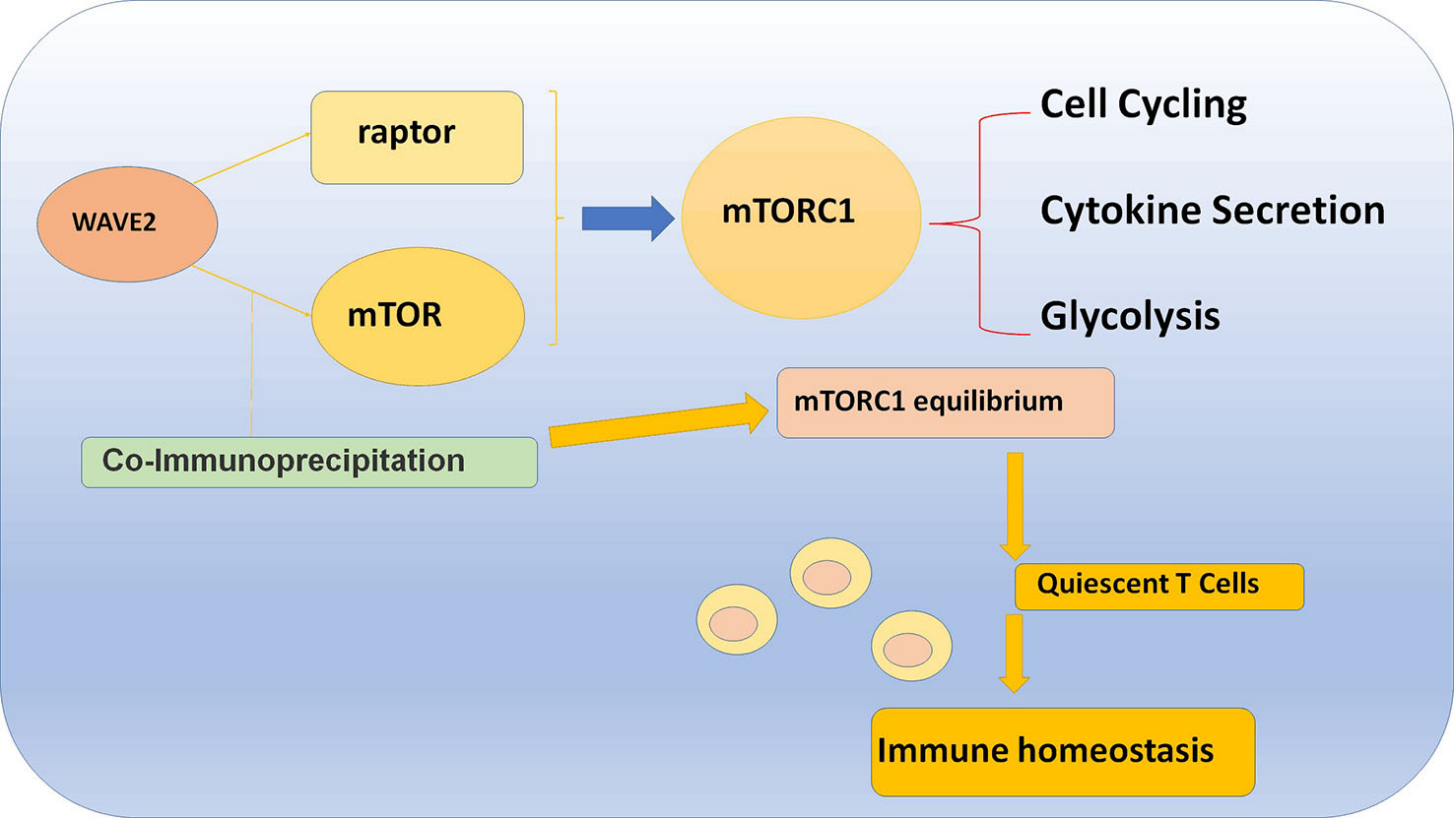
WAVE2 maintains T cell homeostasis and function via binding to Raptor and mTOR, which will cause a coimmunoprecipitation and lead to the reduction of the amount of mTOR and Raptor in T cell.

The mechanism is that WAVE2 colocalized with mTOR in the cell perinuclear region and can directly bind to mTOR which will coimmunoprecipitate mTOR in T cells and MYC-tagged mTOR in transfected cells. Furthermore, WAVE2 can also binds to Raptor, and then cause a coimmunoprecipitation, which leads to the reduction of the amount of mTOR and Raptor in T cells. The final result is that WAVE2 can reduce the amount of mTORC1 and consequently cause a reduction in mTORC1 activation. Moreover, in WAVE2 KO condition, it has been tested that there was an increased coprecipitation of mTOR with Raptor which means that WAVE2 actually suppresses the function of mTORC1. Above all, WAVE2 leads to a “mTOR equilibrium” which lays a solid foundation of T cell homeostasis and function (**Figure 4**) (80).

## Raptor regulates T cell metabolism and promotes the differentiation into Th2 cells

According to studies, Raptor participates in naïve T cell-cycle priming and promotes the differentiation into Th2 cells (81). And the deficiency of Raptor inhibits naïve T cells from entering cell-cycle (82). The mechanism is that Raptor-mTORC1 mediates T cell glycolytic and lipogenic programs, which lays the foundation of the entry into the active cell cycle. Furthermore, the glycolytic and lipogenic condition and Raptor-mTORC1 pathway also intimately associated with cell fate decisions (83, 84). It is worth noting that Th2 cell differentiation have the highest rate of glycolysis among all effector T cells (85, 86).

T cell activation requires CD98 and CD71 (an iron intake receptor), which Raptor KO mice showed a decreased expression of, meaning Raptor mediates the cell-cycle required nutrient uptake. Further studies also demonstrated that the deficiency of Raptor inhibits the expressions of cell-cycle-related genes and the production of cell-cycle-related proteins such as cyclins D2 and E, CDK2, CDK4 and CDK6. Thus, Raptor regulates the cell-cycle machinery (81). Interestingly, it should be noted that Raptor is not essential for T cell proliferation when proliferation has already begun, which means that Raptor-mTORC1 signaling pathway plays a minor role in the subsequent cell division process.

Raptor can also mediate T cell metabolism and then indirectly regulates the proliferation of T cells. Hk2, Ldha, and Tpi1 are glycolytic enzymes, and the expression levels of their messenger RNA are decreased in Raptor KO T cells. What is more, the expression of c-Myc, which is an important transcription factor of T cell glycolysis (87) is decreased in Raptor KO T cells. Studies also concluded that Raptor can help T cells’ interactions with CD28 and TCR stimulation signals and then mediates the production of T cell proliferation-related cytokines including IL-4 and IL-2, which then finally affects the proliferation of T cells. Studies also concluded that the role of Raptor in promoting the differentiation into Th2 cells irrelevant to Rheb, mTORC2, or AKT, which are mTORC1 up-stream effector molecules (81, 88, 89).

## Raptor mediates Th 1 and Th 17 cell differentiation via binding to mTOR

The mechanism of cell differentiation is very complex and often influenced by multiple signaling molecules in complex signal transduction pathways (90). In Th 1 cell differentiation process, IL-12 and IFN-γ stimulate naïve T cells to express T-bet and STAT4, which mediates naïve T cell transform to Th1 cells (91, 92). There are also many factors that regulate Th17 cell differentiation. What has been known clearly is that IL-6 and transforming growth factor (TGF)-β can upregulate the level of retinoic acid-related orphan receptor γt (RORγt), which subsequently promotes the conversion of naive T cells to Th17 cells and initiate CD4+ T cell differentiation process (93–95). Furthermore, phloretin is a dihydrochalcone structural flavonoid compound, which possesses many bioactive characteristics and mediates the AMPK signal pathway and consequently regulates the Th17 cell differentiation balance (96). Th17 cell differentiation is also influenced by glycolysis metabolic reprogramming. Pyruvate Kinase M2 (PKM2) is a glycolytic enzyme that translocates into the cell nucleus and activates STAT3 to promote Th17 cell differentiation (97). Moreover, in a STAT3-dependent way, Th17 cells are stabilized by IL-21 and IL-23 according to a related study (98).

Arctium lappa L (ALF) belongs to traditional Chinese medicine, which is used in treating inflammatory disorders, rheumatic pain and even fever. Arctigenin is a component of ALF that may be valid in treating ulcerative colitis via inhibiting Th 1 and 17 cell functions (99, 100). The mechanism of this traditional Chinese medicine is that it suppresses Th cell differentiation. In various CD4+ T cells, Arctigenin can selectively inhibit the activation of Th1 and Th17 cells (especially Th17 cells) by disturbing the stability of the mTOR1 complex. Arctigenin suppresses the formation of mTORC1 and the activation of the mTORC1 pathway (101) that further affects downstream molecules. In previous research, rapamycin blocks the combination of Raptor and mTOR (102, 103). It has been proven that Arctigenin does not interfere with the production of mTOR and Raptor, but also suppresses the binding of Raptor and mTOR, which consequently suppresses the phosphorylation of the mTORC1 signaling pathway downstream molecules such as p70S6K and RPS6 (101). Meanwhile, the overactivation of Raptor and mTOR can be relieved by the function of Arctigenin. This fully demonstrates the role of Raptor in Th17 cell development.

In addition, Raptor also regulates Th1 cell differentiation and function via other unknown mechanisms. Researchers have found that the decreased Th1 T cell response in the s.c. TME was observed in tumor-bearing Raptor conditional knockout (cKO) mice while the detailed mechanism still requires further research (104).

Ulcerative colitis (UC) is known to be a chronic nonspecific colonic mucosa inflammatory disorder (105). Although there is much debate about the cause of UC, more and more evidence demonstrates that the mechanism is an innate autoimmune response caused by Th1 and Th17 cells subsets and Treg cells (106, 107), which means that Raptor may be a new target of intestinal inflammation therapeutic strategy with strong clinic and therapeutic possibility (108–110).

## Raptor regulates γδ T1 and γδ T17 cell differentiation and function

Like αβ T cells, γδ T cells are innate-like T cells that are essential for fighting infections and tumors (111–115). Though the number of them is far less than αβ T cells, they play an irreplaceable role in the immune function. Raptor not only mediates γδ T cell differentiation, but also regulates their immune functions. The regulatory effect of Raptor on the differentiation of γδ T cells is also mainly through the regulation of cytokine secretion. Interleukin-17 (IL-17)–producing γδ T (γδT17) and interferon-γ (IFN-γ)–producing γδ T (γδT1) cells have different metabolic requirements with being more dependent on glycolysis and phosphorylation respectively (116). Other researchers also found that CD27 is a unique regulator of γδ T1 and γδ T17 cell development. According to the studies, CD27+ γδ T cells will transform into γδ T1 cells, while the other type would differentiate into the γδ T17 cells (117). Further studies have demonstrated that Raptor-mTORC1 can even regulate T cell glycolysis to guide the direction of differentiation (118).

In a study, Raptor and rictor gene knockout mice were used to quantify γδ τ1 and γδ T17 cells. They found that the rate of γδ τ1 and γδ T17 cells were notably decreased in Raptor KO mice, while only the γδ T17 cell rate decreased in rictor KO cells. This phenomenon indicate that Raptor mediates both γδ T1 and γδ T17 cell differentiation, while rictor only participates in γδ T17 cell differentiation (118). Additionally, they further found that mTORC1 promotes IL-17 expression in γδ T cells (118).

Also, in a previous study, it was demonstrated that γδ T cells provide a cytokine environment, such as IFN-γ, NKG2D, to regulate immunity (112, 119). The researchers found that the deficiency of Raptor in γδ T cells leads to a decreased IFN-γ and NKG2D expression. Consequently, the immunological effector capacity of Raptor KO γδ T cells is significantly decreased (118).

## The role of Raptor in NK cells

Natural killer cells (NK cells) are an integral part of the body’s immune system. Derived from hematopoietic stem cells, they mainly focused on antivirus-infection and tumor cell elimination. Moreover, NK cells are particularly involved in hypersensitivity reactions and autoimmune diseases (120). As for NK cells, Raptor can participate in regulating the early reprogramming process mediated by IL-15R indirectly (121). IL-15 has already been proved to be crucial for NK cell development and effector functions by binding with IL-15Rα (CD215) (122–124), and IL-15R can initiate the PI3K-p110δ/p85α in NK cells, which is a upstream signaling pathway and can trigger mTORC1 pathway (125). Studies have found that Lack of Raptor (mTORC1) resulted in decreased quantity of mature NK cell. Furthermore, Raptor-mTORC1 is required for the expression of effector molecules such as Eomesodermin (Eomes) and plays critical roles the transition from CD27 single-positive (SP) to double-positive (DP) NK stage. Additionally, reduced DP and CD11b SP NK cell populations can be observed in RaptorKO mice (126).

The differentiation process of NK cells includes many stages including common lymphoid progenitor stage (CLP stage), NK progenitor stage (NKp stage), immature NK stage (iNK stage), and mature NK stage (mNK stage) (127). Moreover, iNK cells can be grouped into three subsets by the expression of cell surface markers including CD27 and CD11b. The subsets are: CD27− CD11b− (DN, double negative, also refers as iNK stage), CD27+ CD11b−(single CD27 positive), CD27+ CD11b+ and CD27− CD11b+(double positive and single CD11b positive). The expression of CD11b marks the maturation of NK cells, so researchers put CD27+CD11b+ and CD27−CD11b+ subsets into one category (127). In some studies, it has been found that the deficiency of Raptor causes a blockage between the CD27+CD11b- stage and CD27+CD11b+ stage (128). Researchers also found that the development of NK cells are both regulated by mTORC1 and mTORC2. By generating Rptor^fl/fl^/CD122^Cre/+^and Rictor^fl/fl^/CD122^Cre/+^ mice to delete Raptor and Rictor, respectively, and comparing the phenotype of NK cells in Mtor^fl/fl^/CD122^Cre/+^ mice, researchers demonstrated that the absence of Raptor suppressed the differentiation process of immature NK cells (CD27+CD11b−) to mature NK cells while the absence of Rictor limited the differentiation of CD27−CD11b− early immature NK cells. Moreover, the specific deletion of Raptor can affect the process of NK cell terminal differentiation (129, 130).

Additionally, mediating factors such as E4 promoter-binding protein 4 (E4BP4), Eomesodermin (Eomes), T-bet as well as Tsc1 all mediate NK cell development and maturation (121, 131, 132). As have been demonstrated by other studies, E4BP4 and Eomes can maintain IL-15, which is of great importance to NK cell differentiation (133). The interaction between NK cells and IL-15 depends on the level of CD122 expressed on the NK cell surface (129, 134).

**Figure 5:**
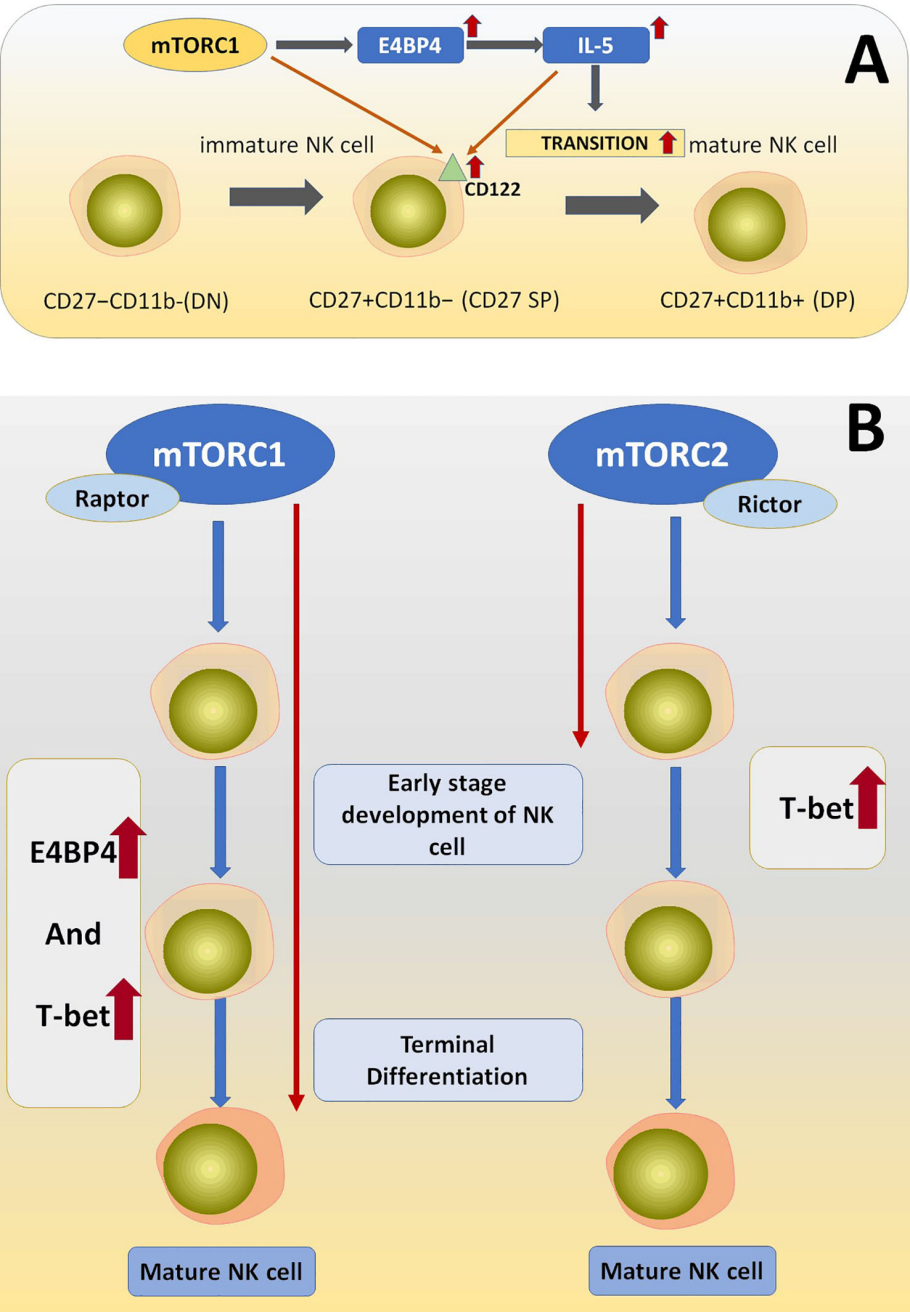
**(A)** The deficiency of Raptor downregulated the amount of CD122 on the NK cell surface. Raptor-mTORC1 signaling pathway play its role in NK cell differentiation via downstream effectors including E4BP4, T-bet, and IL-15, which is essentially needed during NK cell differentiation process. **(B)** mTORC1 is essential for early and late-stage development of NK cells via regulating E4BP4 and T-bet, while mTORC2 is only necessary for very early NK cell development through regulating T-bet.

After examining and comparing the expression condition of E4BP4, Eomes, and T-bet in Rptor^fl/fl^/CD122^Cre/+^and Rictor^fl/fl^/CD122^Cre/+^ mice it was found that Eomes and T-bet were less expressed in Raptor KO NK cells, suggesting that Raptor plays an important role in inducing the production of E4BP4 and T-bet and consequently mediating NK cell early and late-stage differentiation (129) (**Figure 5**).

As for the terminal maturation process of NK cells, researchers generated Rptor^fl/fl^/Ncr1-Cre^Tg^ and Rictor^fl/fl^/Ncr1-Cre^Tg^ mice. After comparing the amount and related subset phenotypes of NK cell (CD27+CD11b+ and CD27−CD11b+) between Mtor^fl/fl^/Ncr1-Cre^Tg^ and Rptor^fl/fl^/Ncr1-Cre^Tg^ and the conclusion is that mTORC1(Raptor) is a key molecule for the terminal differentiation stage of NK cells (129).

## The role of Raptor in iNKT cells

Invariant Natural killer T cells (iNKT) are a unique lymphocyte lineage that are also regarded as CD1d-restricted cells. With the help of CD1d and TCR, iNKT cells can identify glycolipid antigens are not identified by other kinds of T cells (135–138). iNKT cells are regarded as typeINKT cells for their unique TCR α chain, which is encoded by Vα14Jα18 and Vα24Jα18 in mice and humans, respectively, while the β chain of the TCR is not invariant (139–141). Though the amount of iNKT cells is small compared to other types of T cells, iNKT cells are essential in cancer, autoimmunity, infection, allergy and even obesity (142–145). Mature iNKT cells can participate in innate immunity and are essential for adaptive immunity, which also have interactions with B cells (146). INKT cells play a part by producing cytokines such as IL-4, IL-17, IFN-γ and TNF-α after stimulation by the synthetic ligand α-galactosyl ceramide(α-GalCer) (147–149). α-GalCer is separated from the marine sponge Agelas mauritianus, and it can stimulate and activate the cytotoxic capacity of iNKT cells (150, 151). Raptor’s role in NKT cells is similar to that of B cells in that it controls development and function, and just like B cells, Raptor is more critical for cell differentiation than function. It affects both the level of CD molecules expressed on the NKT cell surface and the production of various cytokines, which play roles in signal transduction. Many scientists have been working in this field and this review summarizes their important findings.

iNKT cells can be grouped into categories according to differentiation stages represented by the expression levels of the surface markers CD24, CD44 and CD161 and they are grouped as follows: (CD24+CD44-CD161-), (CD24-CD44–), (CD24-CD44+CD161-), and (CD24-CD44+CD161+) named stage 0 to 3 in turn. Moreover, the expression of CD161 represents the maturation of iNKT cells (138, 140). The differentiation process and function of iNKT cells can be mediated by several transcription factors like RORγt, T-bet, Gata3, and promyelocytic leukemia zinc finger (PLZF) (138, 152–154), which have direct or indirect relationships with Raptor. The quantities of iNKT cells in different stages changed significantly in Raptor KO mice compared to the WT group. Specifically, the number of iNKT cells in stage 0 and 1 is within the normal range, while the number of iNKT cells in stage 2 and 3 is significantly decreased, indicating that Raptor-mTORC1 is needed during the stage 2 transformation and is essential in iNKT cell maturation (155, 156). Also, by further comparing the number of iNKT cells in stage 2 and 3, it can be concluded that Raptor also mediates the differentiation process of iNKT cells from stage 2 to 3.

As has been proven, PLZF is a crucial mediator of iNKT cells (157), and according to some studies, Raptor contributes to the localization of PLZF into the iNKT cell nucleus. The deficiency of Raptor suppresses the ability of PLZF to access differentiation-related gene promoters of iNKT cells (156), which causes a differentiation blockage between stage 1 and 2. This may be one of the reasons for the phenomenon above.

Furthermore, injecting mice with α-GalCer activates iNKT cells and leads to severe hepatitis caused by the overexpression of TNF-α (158, 159). However, in Raptor KO iNKT cells, this phenomenon is absent, which reveals that Raptor participates in regulating the production of TNF-α in iNKT cells. The expression levels of other cytokines such as IL-4 and IFN-γ decreased and the proliferation of iNKT cells was also abolished in Raptor KO mice after stimulating iNKT cells (156).

The deficiency of Raptor also causes reduced iNKT1, increased iNKT2, and normal iNKT17 cell ratios in mice. The explanation may be that Raptor can regulate the production of transcription factors of iNKT cells, although the deeper mechanism still needs to be explored (155, 156, 160).

## Conclusion and future outlook

Raptor is a core component of mTORC1 and plays an essential and complex role in the differentiation processes and functions of lymphocytes. However, there are conflicting results on Raptor‘s role in regulating immune processes, which is specifically seen in studies using Raptor knockouts. With further technical and scientific advancements, researchers will be able to explore deeper into the specific roles of Raptor. The most current studies on Raptor are based on the findings of new phenomena, but none are linked to metabolic disease models such as gastric cancer, colon cancer, diabetes, etc. Since mTORC1 plays a role in the proliferation and metastasis of cancer cells, the regulation of Raptor in the proliferation and differentiation of cancer cells may be a direction worth exploring in the future. Finally, Raptor related studies are being conducted in mouse models, which have been limited to early stages of lymphocytes that only involve a few studies. In short, there is still a long way to go from basic medical theory to clinical application, and it is often very difficult to transition from mouse models to humans, thus if Raptor is to be used as clinical medicine, more efforts need to be made in its research. This review summarizes the current studies on the effects of Raptor on lymphocytes and aims to help researchers find new directions for clinical discovery.

## Author contributions

JT wrote the article. LY, FG, HM, NC, LJ, KB, MK, SH, PL, JL, HZ, and CH revised the draft. ZZ and CL organized and revised the draft. All authors contributed to the article and approved the submitted version.
